# Expectant Management of Severe Preeclampsia in Advanced Maternal Age With Multiple Risk Factors: A Case Report

**DOI:** 10.7759/cureus.79600

**Published:** 2025-02-24

**Authors:** Mohamad Zakour Khadari, Hadzliana Zainal, Nur Aizati Athirah Daud, Abubakar Sha'aban

**Affiliations:** 1 Department of Clinical Pharmacy, School of Pharmaceutical Sciences, Universiti Sains Malaysia, Gelugor, MYS; 2 Department of Population Medicine, School of Medicine, Cardiff University, Cardiff, GBR

**Keywords:** expectant management, gestational hypertension, maternal and perinatal outcomes, maternal morbidity, severe preeclampsia

## Abstract

The management and outcome of severe preeclampsia are outlined in this case report of a 42-year-old multiparous woman presenting with multiple risk factors, including maternal obesity, advanced maternal age, gestational diabetes, and a significant interpregnancy interval of over eight years.

The patient underwent expectant management for close monitoring of her pregnancy. However, due to the emergence of severe preeclampsia symptoms, the decision was made to deliver the baby at 29 weeks of gestation via classical cesarean section and bilateral tubal ligation. The neonate, weighing 1070 g, was born with an Apgar score of 6 and was subsequently admitted to the neonatal unit for severe prematurity.

This case highlights the importance of individualized care plans tailored to patients with multiple risk factors and underscores the proactive management strategies essential for achieving favorable delivery outcomes.

## Introduction

Pregnant women who develop elevated blood pressure (BP) after the 20th week of pregnancy and who do not exhibit proteinuria or other symptoms of organ damage are referred to as having gestational hypertension. Pregnancy-induced hypertension (PIH) is another term for this ailment, and it usually resolves after delivery. Although the precise cause of gestational hypertension is unknown, it is thought to be a result of a mix of genetic, environmental, and immunological events [1,2].

Although gestational hypertension's prevalence varies throughout populations, it is estimated to impact 5-10% of all pregnancies. Preeclampsia or eclampsia, which can have devastating repercussions for both the mother and the fetus, is more likely to occur in women with gestational hypertension [3].

Several nations and areas in Asia have various rates of gestational hypertension prevalence. Although there are no details on the precise frequency of this ailment in many regions of Asia, the evidence that is currently available indicates that it is a serious public health concern throughout the region. Gestational hypertension exhibits higher prevalence rates in certain countries. This may be due to various causes, including increasing maternal age, obesity, and dietary and lifestyle factors [4].

Severe preeclampsia, a distinctive hypertensive disorder of pregnancy, presents intricate challenges that warrant careful consideration in the context of advanced maternal age and coexisting medical conditions.

The prevalence of severe preeclampsia in Malaysia is reported at approximately 1.6% [5]. Despite its rarity, the gravity of this condition is emphasized by the maternal and fetal complications it poses.

Expectant management is a medical strategy involving carefully observing a patient over an extended period rather than taking an immediate medical procedure. This approach relies upon considerations such as the patient's preferences, the severity of the condition, and the delicate equilibrium between associated risks and benefits.

The primary objective of this technique is to circumvent unessential interventions while maintaining it as an accessible standby procedure, poised to be implemented should the need arise [6].

In Malaysian medical practice, the prevailing approach for managing severe preeclampsia cases entails expectant management throughout the remaining duration of the pregnancy. Expectant management aims to mitigate adverse complications and optimize maternal and fetal outcomes [7].

According to established guidelines, the expectant management of severe preeclampsia may necessitate the administration of magnesium sulfate, along with antihypertensive medications, coupled with vigilant monitoring of organ function. Notably, the utilization of magnesium sulfate has been correlated with enhanced maternal outcomes [8].

This case report provides a detailed examination of the diagnostic journey and management strategies employed in addressing severe preeclampsia in a 42-year-old pregnant woman. Notably, the patient carries additional complexities, including maternal obesity with a BMI of 38.0, an interpregnancy interval (>8 years), and a diagnosis of gestational diabetes and gestational hypertension.

The rising trend of pregnancies in women of advanced maternal age prompts a critical examination of associated risks, particularly when compounded by metabolic factors. Our patient's case serves as an illuminating illustration of the convergence of advanced maternal age, maternal obesity, gestational diabetes, and gestational hypertension in the manifestation of severe preeclampsia.

The patient, under care for gestational diabetes with metformin and for gestational hypertension with methyl dopa and labetalol, experienced elevated BP episodes during routine antenatal visits. The culmination of these factors became evident during her last visit, where her BP readings reached an alarming 177/140 mmHg, in addition to the symptoms of lethargy, fatigue, and visual disturbance, underscoring the urgency and complexity of the clinical scenario.

Through this case, we aim to contribute valuable insights into the clinical intricacies, diagnostic challenges, and therapeutic decisions encountered when managing severe preeclampsia in the unique context of advanced maternal age, maternal obesity, gestational diabetes, and gestational hypertension. As obstetric care continually evolves, the experiences shared in this report aspire to provide practical considerations for healthcare professionals navigating the delicate balance of high-risk pregnancies.

## Case presentation

Overview

This case report presents a unique presentation of severe preeclampsia in a pregnant 42-year-old with a complicated medical background. The patient, a single mother whose last childbirth was more than nine years ago (last childbirth (LCB) > 8 years), had previously been diagnosed with maternal obesity, gestational hypertension, and gestational diabetes.

The clinical scenario unfolded during a routine prenatal check-up at Hospital Seberang Jaya, Malaysia, where the patient exhibited elevated BP readings despite her previous treatment for gestational hypertension, which is composed of methyl dopa and labetalol. Subsequent measurements revealed persistently high BP levels accompanied by symptoms of fatigue and visual disturbances, and these clinical features led to a diagnosis of severe preeclampsia.

Medical management decisions were crucial in light of the severity of the condition. A decision was made to pursue expectant management in the hospital for the remaining duration of the pregnancy. This decision could spare the patient significant risks, including the potential for eclampsia, stillbirth, and long-term organ damage.

The case underscores the intricate balance between medical management strategies and patient autonomy, particularly in challenging clinical scenarios such as severe preeclampsia. It highlights the importance of individualized care and the need for comprehensive risk assessment in guiding management decisions for pregnant patients with complex medical histories.

Patient history

Obstetric History

The patient is 42 years old and in her 28th week of gestation. The patient is currently in her third pregnancy (gravida 3), having previously experienced two live births (para 2). She has given birth twice before, as shown in Table 1 below.

**Table 1:** Patient’s obstetric history

Year	Weight of the newborn (kg)	Sex	Breastfeed (month per year)
2011	3.6	Boy	3/12
2015	3.2	Boy	1/12

The patient's obstetric history reveals a noteworthy absence of complications during her two preceding pregnancies, indicating an uneventful course without any reported issues or adverse outcomes.

Medical History

The patient reports an absence of any significant medical history, with no documented occurrence of diseases or health conditions. Additionally, there is no noteworthy family history of medical disorders or hereditary conditions.

Current pregnancy

In the current gestational period, the patient presents several notable issues, foremost among them maternal obesity, as indicated by a body mass index (BMI) of 38 kg/m^2^. The patient weighs 90.5 kg, and her height measures 153 cm. This clinical information underscores the presence of a significant risk factor, necessitating careful consideration and management throughout the pregnancy.

Furthermore, the patient is concurrently grappling with gestational diabetes and gestational hypertension, a condition that adds challenge to the current pregnancy. The diagnosis of these two gestational complications necessitates close monitoring and comprehensive management to mitigate potential complications and optimize maternal and fetal health outcomes.

These challenges underscore the imperative for a multidisciplinary approach and vigilant prenatal care throughout the pregnancy.

The patient's most recent blood glucose levels are delineated in Table 2 below.

**Table 2:** Patient's glucose readings

Date	Test	Results	Unit	Reference range
November 23, 2023	Fasting blood sugar	5.4	mmol/L	3.5-6.0
November 23, 2023	2-hour postprandial test	8.8	mmol/L	3.9-6.7
October 30, 2023	Fasting blood sugar	5.7	mmol/L	3.5-6.0
October 30, 2023	2-hour postprandial test	7.9	mmol/L	3.9-6.7

Nevertheless, the clinical picture is further complicated by the diagnosis of gestational hypertension in the patient. Elevated BP readings were documented during her antenatal visits, as illustrated in Table 3 below.

**Table 3:** Patient’s blood pressure readings

Date	Blood pressure readings	Pulse rate
December 14, 2023	177/140 mmHg	90
November 26, 2023	156/111 mmHg	90
November 23, 2023	141/99 mmHg	91
November 19, 2023	107/86 mmHg	71
November 17, 2023	162/104 mmHg	80
November 2, 2023	134/80 mmHg	73

During the most recent antenatal examination, the patient exhibited an elevated BP reading of 177/140 mmHg, indicative of a considerable increase. The patient presented with marked impairment, characterized by a notable lack of energy, a pallid face, visual disturbance, and drowsiness, to the extent that even verbal communication proved challenging. The gynecologist rendered a diagnosis of severe preeclampsia based on the clinical presentation; subsequently, the decision was favorable to admit the patient to the hospital for close monitoring.

Diagnostic investigations

The laboratory tests of the patients are shown in Table 4.

**Table 4:** Patient’s laboratory results

Test	Results	Reference range
White blood cells	12.8	4.0-11.0 × 10^3/uL
Red blood cells	4	3.8-5.8 × 10^6/uL
Hemoglobin	10.7	11.5-16.5 g/dL
Hematocrit	31.4	37.0-47.0%
Mean corpuscular volume	78.5	76.0-96.0 fL
Mean corpuscular hemoglobin	26.8	27.0-32.0 pg
Mean corpuscular hemoglobin count	34.1	30.0-35.0 g/dL
Platelet	250	150-400 × 10^3/uL
Lymphocyte%	16.2	15-45.8%
Neutrophil%	79.8	43.7-77.1%
Lymphocyte count	2.1	1.5-4.0 × 10^3/uL
Neutrophil count	10.2	2.0-7.5 × 10^3/uL

Diagnosis

In light of the patient's recent BP measurements, notably registering at 177/148 mmHg during the last visit, coupled with reported physical symptoms such as headache and fatigue, visual disruption, and in consideration of the concurrent presence of multiple risk factors including advanced maternal age, maternal obesity, gestational hypertension, and gestational diabetes, the clinical diagnosis indicates severe preeclampsia with a high risk of stillbirth. Consequently, there is an imperative for intensive and direct medical oversight daily for the remaining pregnancy period to address the critical nature of the patient's condition.

Treatment

In light of the diagnosed severe preeclampsia and the critical nature of the patient's condition, admission to the ward is strongly advised for continuous monitoring and comprehensive assessment throughout the remaining pregnancy period. An electrocardiogram (ECG) is also recommended post-admission to evaluate cardiovascular function. The following medications listed in Table 5 are continued to manage the patient's current medical conditions.

**Table 5:** Patient’s current medication BID: twice per day; TDS: three times a day; OD: once a day

Medication	Dose	Frequency
Methyldopa	500 mg	TDS
Labetalol	100 mg	TDS
Aspirin	150 mg	OD
Metformin	500 mg	BID

The concurrent administration of labetalol and methyldopa represents a viable therapeutic approach that healthcare providers may contemplate for the management of hypertension in pregnant individuals afflicted with preeclampsia. Both pharmaceutical agents are commonly employed in regulating elevated BP during gestation, each possessing distinct mechanisms of action [5].

## Discussion

Severe preeclampsia, an uncommon but critical pregnancy complication, demands careful consideration, particularly when accompanied by other risk factors. Our case involves a 42-year-old pregnant woman with a BMI of 38.0, LCB >8 years, gestational diabetes, and gestational hypertension, which developed into severe preeclampsia, marked by a hypertensive crisis (177/140 mmHg), fatigue, and visual disturbance during the patient's latest antenatal visit.

Each one of the patient's risk factors predisposes her to a higher risk of severe preeclampsia. Starting with the patient's age, pregnant women >40 years are at higher risk of developing preeclampsia [9,10]. Pregnant women with maternal ages between 30 and 50 years old have a higher proportion of developing preeclampsia compared to women with younger maternal ages despite them having additional risk factors [11].

Moreover, obesity is an additional risk factor for emerging preeclampsia. Incidences of preeclampsia are four times higher in obese pregnant women compared to non-obese pregnancies [12]. Maternal obesity itself is a risk factor for preterm birth, stillbirth, gestational hypertension, gestational diabetes, and other cardiovascular complications [13].

The risk of preeclampsia is known to be higher in nulliparous compared to multiparous. However, this risk seems to be increasing in multiparous when their LCB, or as referred to as interbirth interval (IBI) has occurred older than 5 years ago (LCB > 5 years), even if those women have no history of preeclampsia [14]. 

The patient in this case report has had all of these risk factors, which necessitated the medical team to put her through expectant management in the hospital with all standby options.

Expectant management includes treatment according to the patient's condition and prophylactic measures, including administering magnesium sulfate and dexamethasone.

Furthermore, it entails biweekly analyses of blood and urine, frequent BP assessments (3-4 times daily), and clinical evaluations to discern any indicative symptoms signaling the progression of preeclampsia [15].

Moreover, the protocol involves vigilant monitoring of liver function, renal function, and coagulation system factors to promptly identify potential organ dysfunction [16].

Our patient has received a regimen to control her BP, which included methyldopa 500 mg three times a day (TDS) and labetalol 100 mg TDS, in addition to metformin to manage her gestational diabetes.

The expectant management aims to monitor the patient until her due date and provide the necessary treatment. However, the patient's condition hasn't shown signs of improvement despite being admitted to the ward where she received the required treatment for her condition; her BP readings continued to register high levels. Considering the patient's age and other risk factors, the medical decision was made to perform an early cesarean section to stabilize her condition and assess the fetus.

On week 29 of gestation, the patient underwent a classical cesarean section and bilateral tubal ligation. The delivery outcome was a 1070-gram female baby with an Apgar score of 6 at the first minute. The neonate was admitted to the neonatal unit for monitoring due to severe prematurity.

The current report has shed light on a preterm delivery following a severe preeclamptic case for an advanced maternal-age woman with multiple risk factors. The medical intervention has succeeded in saving both the pregnant woman and her fetus through an immediate medical procedure in the 29th week of gestation.

Identifying high-risk patients during antenatal visits and providing suitable management for them is crucial in terms of sparing the pregnant mother and her fetus various complications that might include the death of either of them.

## Conclusions

In conclusion, this case report highlights the complexities of managing severe preeclampsia in the presence of multiple risk factors. It emphasizes the importance of individualized, multidisciplinary care in high-risk pregnancies and serves as a basis for further research to improve outcomes in similar cases.
